# Compact wideband implantable antenna for wireless capsule endoscopy application in the 2.45 GHz ISM band

**DOI:** 10.1038/s41598-025-15591-8

**Published:** 2025-08-20

**Authors:** Archana Mohan, Niraj Kumar

**Affiliations:** School of Electronics Engineering, Vellore Institute of Technology, Chennai, Tamil Nadu India

**Keywords:** Implantable antenna, Capsule endoscopy, Defective ground structure, Wideband, Link budget, Specific absorption rate (SAR), Biomedical engineering, Electrical and electronic engineering

## Abstract

This paper presents a compact wideband implantable antenna developed for wireless capsule endoscopy (WCE) functioning in the 2.4–2.48 GHz ISM band. The antenna’s miniaturized design is achieved through the integration of E - and L - shaped slots in the circular radiator, a shorting pin and defective ground structure with an inclined T – shaped slot. The final structure has compact dimension of $\:\pi\:\:\times\:\:{3}^{2}\:\times\:\:0.254\:m{m}^{3},$ and the Rogers RO3010 ($\:{\epsilon\:}_{r}=10.2,\text{tan}\delta\:=0.0035$) material serves as both substrate and superstrate. Performance was assessed in homogeneous muscle and heterogeneous human phantoms, and experimentally validated by implanting the device in minced pork. At 2.45 GHz the antenna delivered − 20.8 dBi gain, and a 44.02% impedance bandwidth. The specific absorption rate (SAR) values are recorded as 216.8 W/kg (1 – g) and 30.2 W/kg (10 – g). The study demonstrates that the antenna reliably supports wireless links beyond 10 m, maintaining a 10 dB margin at 2.45 GHz.

## Introduction

Wireless capsule endoscopy (WCE) is a sophisticated diagnostic technique for imaging the gastrointestinal (GI) tract. In recent years, this cutting-edge technology has garnered significant interest because of its noninvasive and painless nature, making it a highly attractive alternative to conventional wired endoscopic procedures. Traditional endoscopy, which remains the highest standard for GI examinations, is often associated with invasiveness and patient discomfort. Additionally, it has inherent limitations, such as its inability to offer a complete view of the small intestine^1–6^. The antenna used in WCE plays a crucial role in transmitting and receiving electromagnetic waves, facilitating communication between the capsule and external equipment. However, forming a dependable wireless connection among an implantable endoscopic antenna and an exterior device remains challenging due to multiple influencing factors^7,8^.

**Fig. 1:**
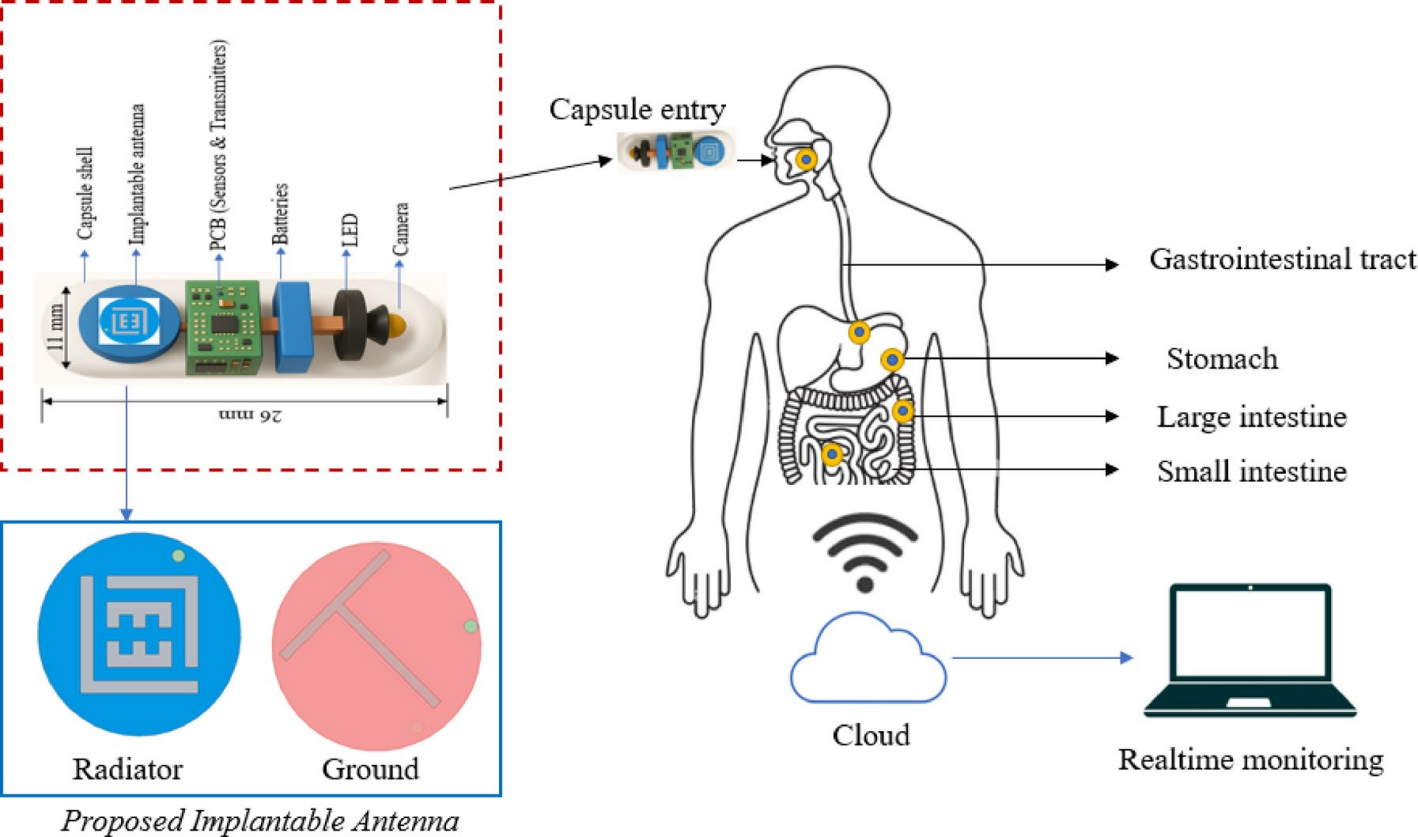
Overview of the proposed wireless capsule endoscopy system.

Current research shows that conformal^9^ and embedded designs are the favored antenna types for capsule endoscopy^10,11^. Figure 1 presents an overview of the wireless capsule endoscopy system. The capsule contains of a biocompatible capsule shell, a miniaturized implantable antenna, electronic circuits that have sensors and communication units, batteries, LEDs and CMOS cameras. The implantable antenna plays a crucial role in facilitating wireless communication, acting as a link between the capsule and external receivers. The endoscope implantable antennas must be compact enough to fit within extremely small integrated circuits. Researchers have proposed various size reduction techniques, such as high permittivity materials, capacitive slots, shorting pins, inductive stubs, spiral – shaped, slot – based, meandering – line radiators^12^, defective ground structures^13–15^ coplanar waveguide structures^16^ and slow wave structures to achieve miniaturization^17–22^.

A compact wideband implantable antenna was proposed in^23^ for capsule endoscopy application at 915 MHz. A rectangular radiator is used for miniaturization. The footprint of $\:7\:mm\:\times\:7\:mm\:\times\:1.6\:mm$ produces a 300 MHz bandwidth. However, the gain and bandwidth are very low for capsule endoscopy application A circularly polarized dual band implantable antenna was proposed in^24^ and antenna footprint was reduced by using open-ended slots in the ground and a meandering line structure in the radiator and the dimensions were $\:6.5\:mm\:\times\:6.5\:mm\:\times\:0.05\:mm$. Symmetrical and asymmetrical slots in the radiator facilitate the generation of circular polarization. The bandwidths of the proposed antenna were 123.5 MHz and 154.4 MHz at 915 MHz and 2450 MHz respectively. The proposed antenna provided very low bandwidth and high SAR values and it makes difficulties in practical implementation. The dual polarized circular shape implantable antenna with meandering ring slots was reported in^25^ at the 2450 MHz ISM band. A pair of meandered ring slots with degenerative modes and pairs of inductive elements is used to obtain dual polarization competence. The proposed meander line antenna has a foot print of $\:\pi\:\:\times\:\:{\left(5\:mm\right)}^{2}\:\times\:1.28\:mm\:,$ and it provides lower bandwidth was 160 MHz. A wideband implantable antenna was previously implemented in^26^ for capsule endoscopy applications. The meandering line structure and rectangular slots in the radiator, defective ground structure and vias were used to reduce the antenna dimensions, and the antenna footprints are $\:\pi\:\:\times\:\:{\left(5\:mm\right)}^{2}\:\times\:0.635\:mm$ at 2450 MHz. The capacity loading technique was used to increase the impedance bandwidth. The proposed antenna provided a bandwidth of 520 MHz at 2450 MHz. However, it stimulates very high SAR value of 1 – g.

An omni directional circularly polarized antenna with a compact size and wideband capabilities at the 2450 MHz ISM band was proposed in^27^. The proposed antenna achieves circular polarization in all directions. The footprint of the proposed antenna $\:\pi\:\:\times\:\:{\left(4.9\:mm\right)}^{2}\:\times\:0.835\:mm$ and it was achieved with the help of arc shaped slits in the patch, parasitic patches and a shorting pin. The proposed antenna delivers an impedance bandwidth of 150 MHz at 2450 MHz. It depicts very low gain and bandwidth, so practically image transmission is very difficult in this bandwidth. A miniaturized implantable antenna with circular polarization was introduced in^28^ at the 2450 MHz ISM band for capsule endoscopy applications. The dimensions of the proposed antenna were $\:\pi\:\:\times\:\:{\left(4mm\right)}^{2}\:\times\:0.889\:mm$ and it was achieved via a circular radiator with chamfered rectangular slots, a curved line in the middle and ground plane contains an X – shaped slot. The proposed antenna provides a 120 MHz bandwidth at 2450 MHz. However, the bandwidth and gain values are very low. A dual layer patch for radiation was proposed in^29^ at the 2450 MHz ISM band for capsule endoscopy applications. Two different types of meandering line structures were used in the proposed antenna and flexible slots are used at the bottom plane. Measuring $\:2.6\:mm\:\times\:\:3\:mm\:\times\:\:0.381\:mm$, the antenna achieved a 148 MHz impedance bandwidth at 2.45 GHz. An ultraminiaturized implantable antenna was proposed in^30^ with semi-circular slots and vias were used for miniaturization and dimensions of the proposed antenna were $\:\pi\:\:\times\:\:{\left(3\:mm\right)}^{2}\:\times\:0.254\:mm$ which provides a 400 MHz bandwidth. Still the above two proposed works are small in size, but the bandwidths are very low so, it restricts its practical implementation. Most previous studies have focused either on external transmission systems or common antenna designs without fully integrating realistic in-body scenarios or addressing the compact size constraints of actual capsules.

In wireless capsule endoscopy applications, miniaturized antennas with broader bandwidth and improved gain are preferred to ensure reliable communication while minimizing tissue absorption. To address this research gap, this work presents a compact, wideband implantable antenna specifically designed for WCE applications operating in the 2.4–2.48 GHz ISM band. The antenna is optimized to function efficiently within human tissue while maintaining a small form factor suitable for capsule integration. With its ultra – compact footprint of $\:\pi\:\:\times\:\:{\left(3\:mm\right)}^{2}\:\times\:0.254\:mm$, the proposed antenna outperforms conventional designs by preserving high radiation efficiency despite its small size. Its performance was validated at a 75 mm depth using both a homogeneous muscle phantom and a heterogeneous humantorso phantom. The antenna and muscle phantom models were designed and simulated using Ansys HFSS 2023 R2, a widely used tool for biomedical electromagnetic analysis. The proposed work provides ultra – wideband of 1050 MHz and − 20.8 dBi gain value. The proposed work has reliable communication capability, maintaining a wireless link margin exceeding 10 dB over distances greater than 10 m, confirming the antenna’s viability for WCE telemetry. This study is structured as follows: Section II covers antenna configuration, Section III examines the antenna’s performance in terms of S_11_, bandwidth, gain, specific absorption rate (SAR), and radiation patterns, Section IV presents the link-budget analysis, which evaluates the efficiency of wireless signal transmission through muscle tissue.

## Antenna configuration

### Simulation environment

**Fig. 2:**
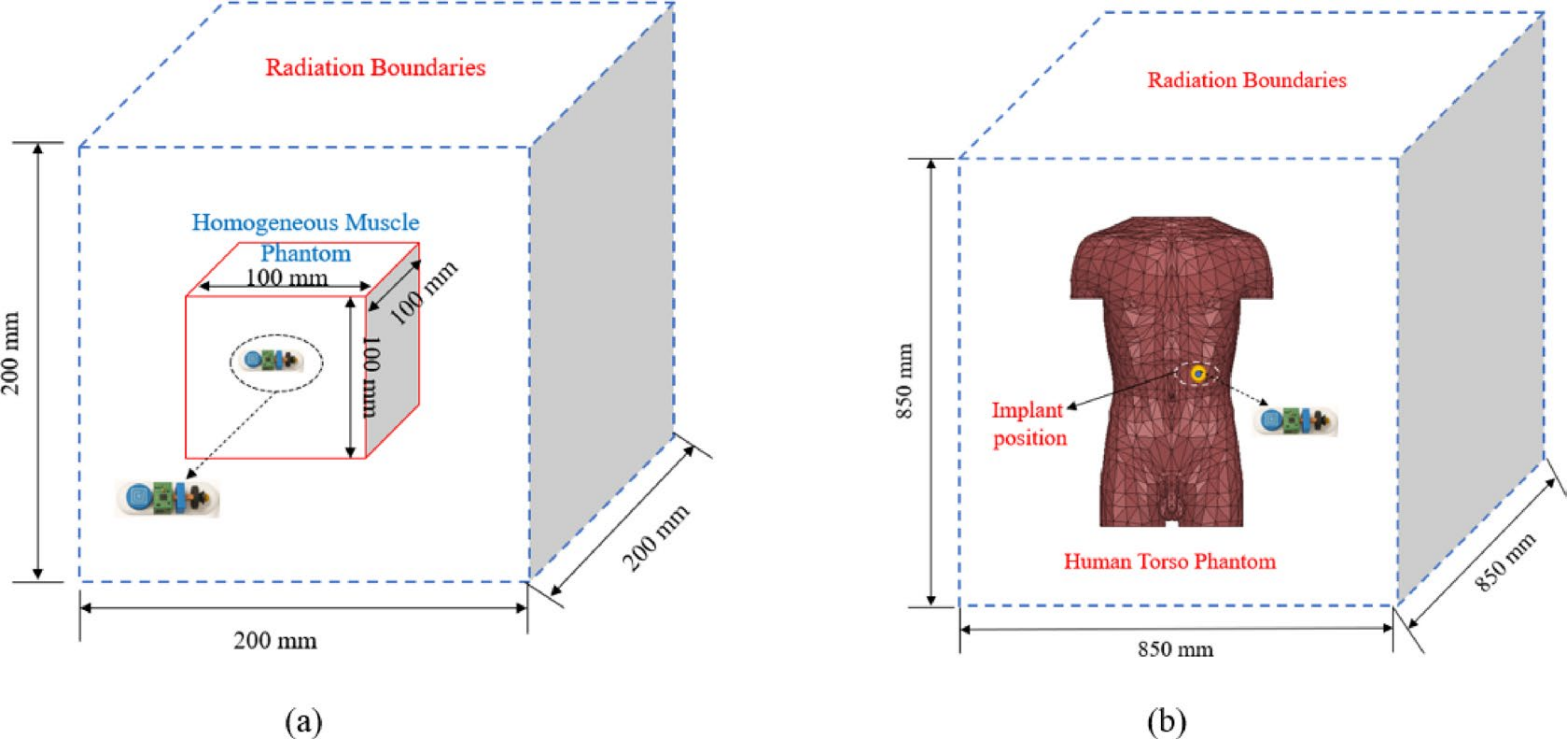
Simulation environments (**a**) homogeneous muscle phantom and (**b**) realistic human torso phantom.

The antenna simulation was conducted using Ansys high-frequency simulation (Ansys HFSS 2023 R2 - https://www.ansys.com/) software via a homogeneous cubical phantom measuring $100~ \times ~100~ \times ~100~mm^{3}$. The antenna was integrated into a wireless capsule endoscopy (WCE) device measuring 26 mm long with a 5.5 mm radius. (Fig. 1). The antenna was positioned 75 mm deep within the phantom, as shown in Fig. 2a. To ensure accurate performance evaluation, the phantom was model to replicate the frequency-dependent dielectric properties of human muscle tissue, specifically at 2.45 GHz. The modelling considers the relative permittivity $\:\left({\epsilon\:}_{r}\right)$ and conductivity $\:\left(\sigma\:\right)\:$to ensure realistic electromagnetic behavior during the simulation as shown in Table 1.

**Table 1:** Properties of gastrointestinal tract tissues at 2.45 GHz.

Phantom	Relative permittivity ($\:{\varvec{\epsilon\:}}_{\varvec{r}}$)	Conductivity $\:\left(\varvec{\sigma\:}\right)\:\left(\varvec{S}/\varvec{m}\right)$
Muscle	52.7	1.74
Large intestine	53.9	2.04
Small intestine	54.4	3.17
Stomach	62.2	2.21

Building on the initial simulation in the homogeneous phantom, we further refined our study using a heterogeneous human torso model in the HFSS to better capture the complexities of real human tissue interactions. In this configuration, the capsule was placed inside the stomach at a 75 mm depth to realistically replicate its interaction with surrounding biological tissues, as illustrated in Fig. 2b.

### Design and optimization of the implantable antenna

**Fig. 3:**
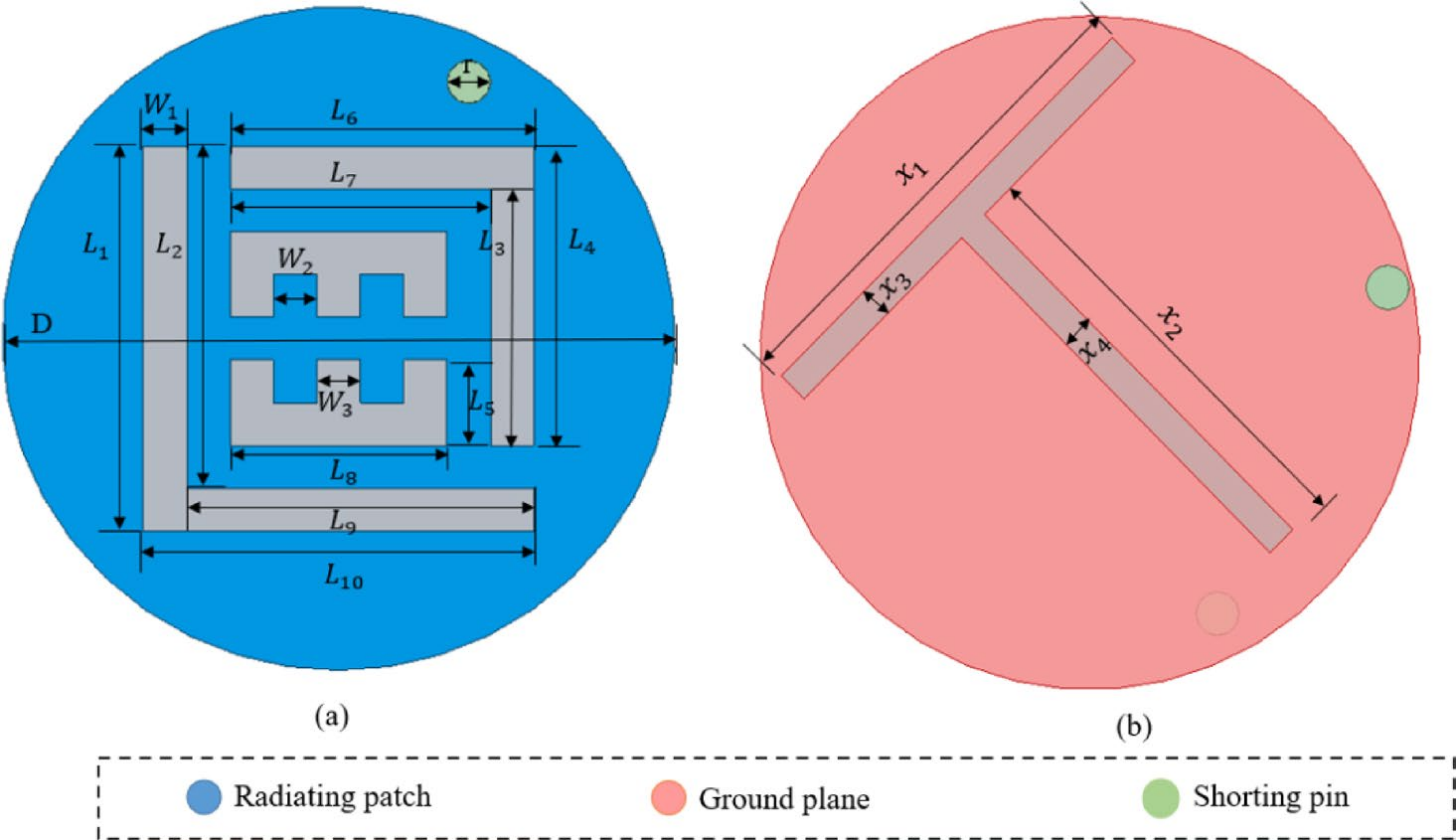
Geometry of the proposed implantable antenna (**a**) top view (**b**) bottom view.

Figure 3 illustrates the proposed implantable antenna from both top and bottom perspectives, with its optimized dimensions are shown in Table 2. The antenna employs a circular patch configuration, excited by a 50 Ω coaxial probe, and incorporates a defective ground structure. To achieve miniaturization, a 0.2-mm diameter shorting pin was incorporated into the design. Additionally, a strategically placed pair of E - and L - shaped slots in the radiator and an inclined T – shaped slot are introduced into the ground to enable 2.45 GHz ISM band functionality while preserving its compact structure. The antenna’s superstrate and substrate are constructed using Rogers RO3010 material, which features a relative permittivity $\:\left({\epsilon\:}_{r}\right)\:$of 10.2, a low loss tangent $\:\left(tan\:\delta\:\right)$ of 0.0035, and a thickness of 0.254 mm. The implantable antenna is enclosed within a capsule shell made of polylactic acid (PLA), which has a relative permittivity $\:\left({\epsilon\:}_{r}\right)$ of 2.8 and a thickness of 0.2 mm. In our design the antenna’s substrate/superstrate are never in direct contact with tissue instead, the entire assembly (including the Rogers layers) is enclosed within a biocompatible PLA shell which comply with ISO 10993 requirements for implantable devices.

### WCE system model

To evaluate the antenna in a realistic operating environment, a Wireless Capsule Endoscopy (WCE) system model was developed. The working principle of the setup is illustrated in Fig. 1. The capsule was made of Polylactic acid (PLA), which is a biodegradable polyester widely used in medical implants. PLA degrades by hydrolysis into lactic acid, a natural metabolite, giving it “favourable biocompatibility and safe degradation products. In vitro tests show PLA is essentially noncytotoxic (e.g., HeLa cells retain > 80% viability in PLA extract test). In vivo, PLA implants provoke only a mild foreignbody reaction. The capsule measures 26 mm in length and 5.5 mm in radius. Typically, deep-tissue capsule devices comprise various components, including implantable antenna, CMOS image sensors, surface-mounted lumped elements (such as capacitors and resistors), light-emitting diodes (LEDs), microcontroller, 35 mAh silveroxide coin cells (CR1025), and 2.4 GHz transceivers.

All the components are organized in a layered stack configuration. Initially the CMOS camera sensors with LED light source after that the pair of stacked 3 V, 35 mAh silveroxide coin cells (CR1025) are housed in a springloaded bracket at the rear. This configuration delivers a stable 6 V supply, regulated down to 3.3 V and 1.8 V rails for the transceiver and microcontroller, respectively. The 2.4 GHz transceiver was connected with the ultra – compact implantable antenna. A biocompatible PLA is 3Dprinted in two halves, which click together around the internal modules. The design was simulated inside a human body phantom, specifically within the gastrointestinal tract, as depicted in Fig. 2.

**Table 2:** Parameters of proposed implantable antenna.

Parameters	Values (mm)	Parameters	Values (mm)
$\:D$	6	$\:{L}_{9}$	3.2
$\:{L}_{1}$	3.6	$\:{L}_{10}$	2
$\:{L}_{2}$	3.2	$\:{W}_{1}$	0.4
$\:{L}_{3}$	2.4	$\:{W}_{2}$	0.4
$\:{L}_{4}$	2.8	$\:{W}_{3}$	0.4
$\:{L}_{5}$	0.8	$\:{x}_{1}$	4.4
$\:{L}_{6}$	2.8	$\:{x}_{2}$	4.1
$\:{L}_{7}$	2.4	$\:{x}_{3}$, $\:{x}_{4}$	0.3
$\:{L}_{8}$	3.6	$\:r$	0.4

**Fig. 4:**
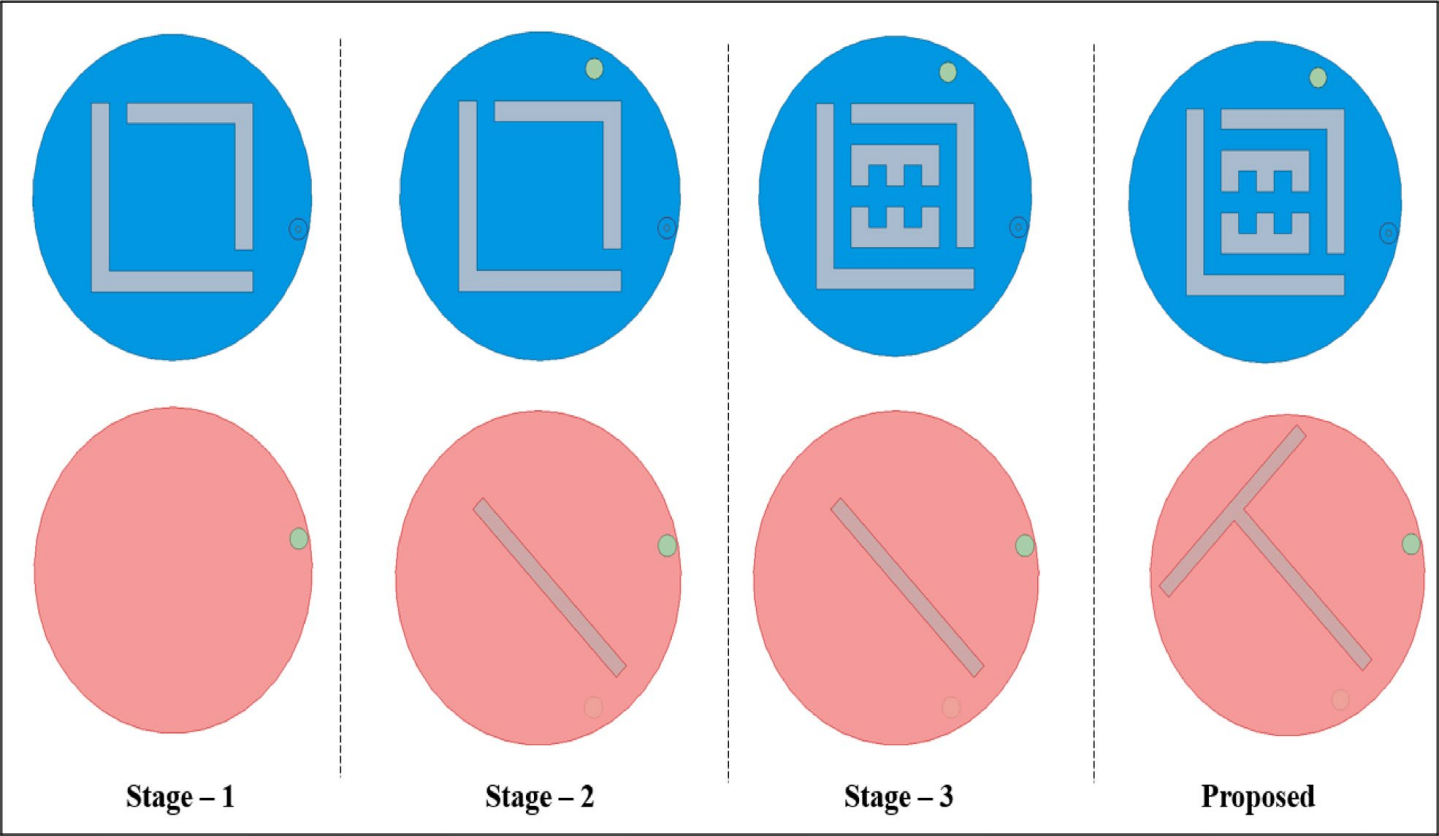
Design stages.

**Fig. 5:**
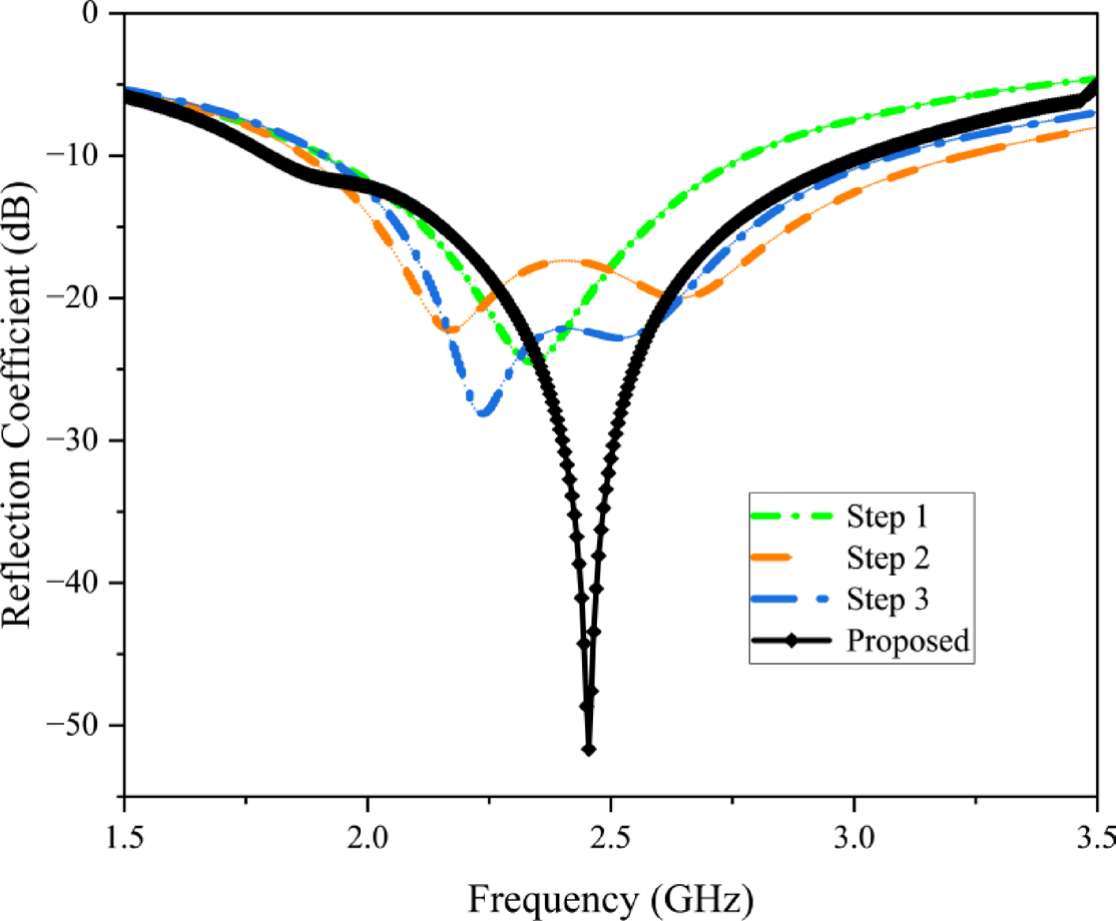
Reflection coefficients of various stages.

The proposed implantable antenna is developed through four key phases, each involving iterative enhancements to optimize performance. A comprehensive overview of these design stages, along with the corresponding reflection coefficient $\:\left({S}_{11}\right)$ comparisons, is presented in Figs. 4 and 5. During the design process, modifications are made to the fundamental patch and ground shape. In the first design phase (Step 1), the antenna consists of a complete circular patch, which is excited through a coaxial probe feed with a pair of L – shaped slots and resonates nearly at 2.15 GHz. In Stage 2, a shorting pin was introduced between the patch and ground, and a rectangular slot was incorporated in the ground structure, consequentially the antenna resonance at two frequencies: 2.1 GHz and 2.6 GHz. In Stage 3, the ground plane was kept the same as that in Stage 2, and in the radiator plane pair of E – shaped slots were added, as a result the antenna resonated at 2.2 GHz. Lastly, an inclined T-shaped slot was added to the ground plane to target the 2.45 GHz ISM band. The finalized antenna achieves a broad bandwidth of 1,220 MHz (1,800–3,020 MHz) and exhibits excellent impedance matching with minimal reflection at 2.45 GHz.

### Parametric examination

The antenna’s performance is highly sensitive to the dimensions and placement of its features, so we performed a parametric study to refine the design. We varied the radiator’s slot width (W_1_), the slot length (x_2_) in the ground structure, changing the location of shorting pin and implantation depth of capsule antenna within the simulation environment, then examined the resulting S_11_ response to identify the optimal configuration.

### Effect of varying the slot width (W_1_)

**Fig. 6:**
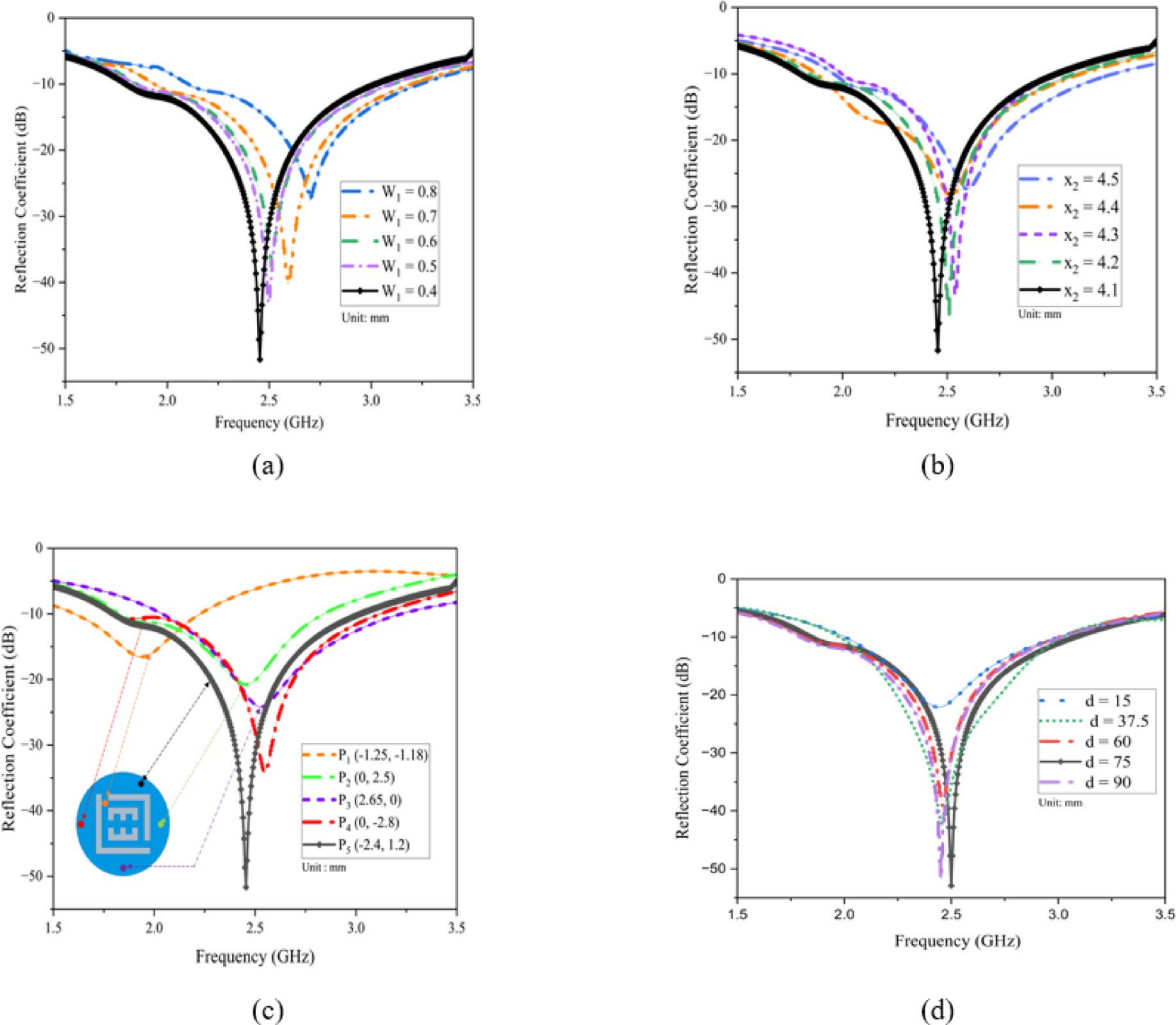
Parametric examination of the proposed antenna (**a**) Effect of varying the slot width W_1_ (**b**) Effect of varying the slot length x_2_ (**c**) Effect of changing the shorting pin location (**d**) Effect of varying the implantation depth.

The slots are integrated into the radiating patch to minimize the antenna’s overall size. The slot length was adjusted between 0.4 and 0.8 mm to assess its effect on the resonant frequency. The analysis revealed that increasing the slot length caused a frequency shift from 2.82 GHz to 2.45 GHz and it is shown in Fig. 6a. This shift highlights the crucial role of the slot dimensions in tuning the resonant frequency, thereby facilitating the miniaturization of the antenna.

### Effect of varying the slot length (x_2_)

Miniaturization was achieved by using a defective ground structure, with a slot length of x_2_ playing a critical role in miniaturization and to achieving the target frequency. A set of simulations was performed with the ground plane slot length (x_₂_) varied from 4.1 to 4.5 mm to evaluate its effect on the reflection coefficient. As a result, the resonant frequency band shifted from 2.65 GHz to 2.45 GHz and it is shown in Fig. 6b. This shift occurs because incorporating slots in the ground plane extends the current path, thereby lowering the resonance frequency.

### Effect of changing the shorting pin location

Shorting pins are commonly used in miniature antennas to lower resonance frequency and shrink size, but their exact placement profoundly affects resonance and impedance. We evaluated five pin locations (Fig. 6c). At P_1_ (–1.25, − 1.18), situating the shorting pin opposite the feed produced a 1.8 GHz resonance with inadequate impedance matching. Relocating the pin closer to the feed at P_2_ (0, 2.5) shifted the resonance to 2.32 GHz. Positioning it at the base of the L-shaped slot at P_3_ (2.65, 0) produced a 2.55 GHz resonance. Moving the pin to P_4_ (0, − 2.8) resulted in a 2.6 GHz resonance. Finally, at P_5_ (–2.4, 1.2), the pin achieved resonance within the target 2.45 GHz ISM band and provided the widest bandwidth.

### Effect of varying the implantation depth

The antenna operates inside the human body, its design must contend with varying tissue environments. Once the capsule is ingested, it travels sequentially through the colon, stomach, small intestine, large intestine, and continuously changing its position. Consequently, an antenna integrated into a capsule endoscope must deliver consistent performance at different depths within the body. Figure 6d presents simulated |S₁₁| results when the distance d between the antenna and the outer peripheral muscle along the Z-axis is set to 15 mm, 37.5 mm, 60 mm, 75 mm, and 90 mm. From Fig. 6d, it is evident that at an implantation depth of d = 75 mm, the antenna resonates at 2.45 GHz with a reflection coefficient of − 54 dB. At depths of 15 mm, 37.5 mm, 60 mm, and 90 mm, the resonant frequency remains close to 2.45 GHz, with the reflection coefficient hovering around − 20 dB and bandwidth also remains constant along all the cases. Therefore, the antenna maintains stable impedance matching across the range of simulated implantation depths.

## Results and discussion

**Fig. 7:**
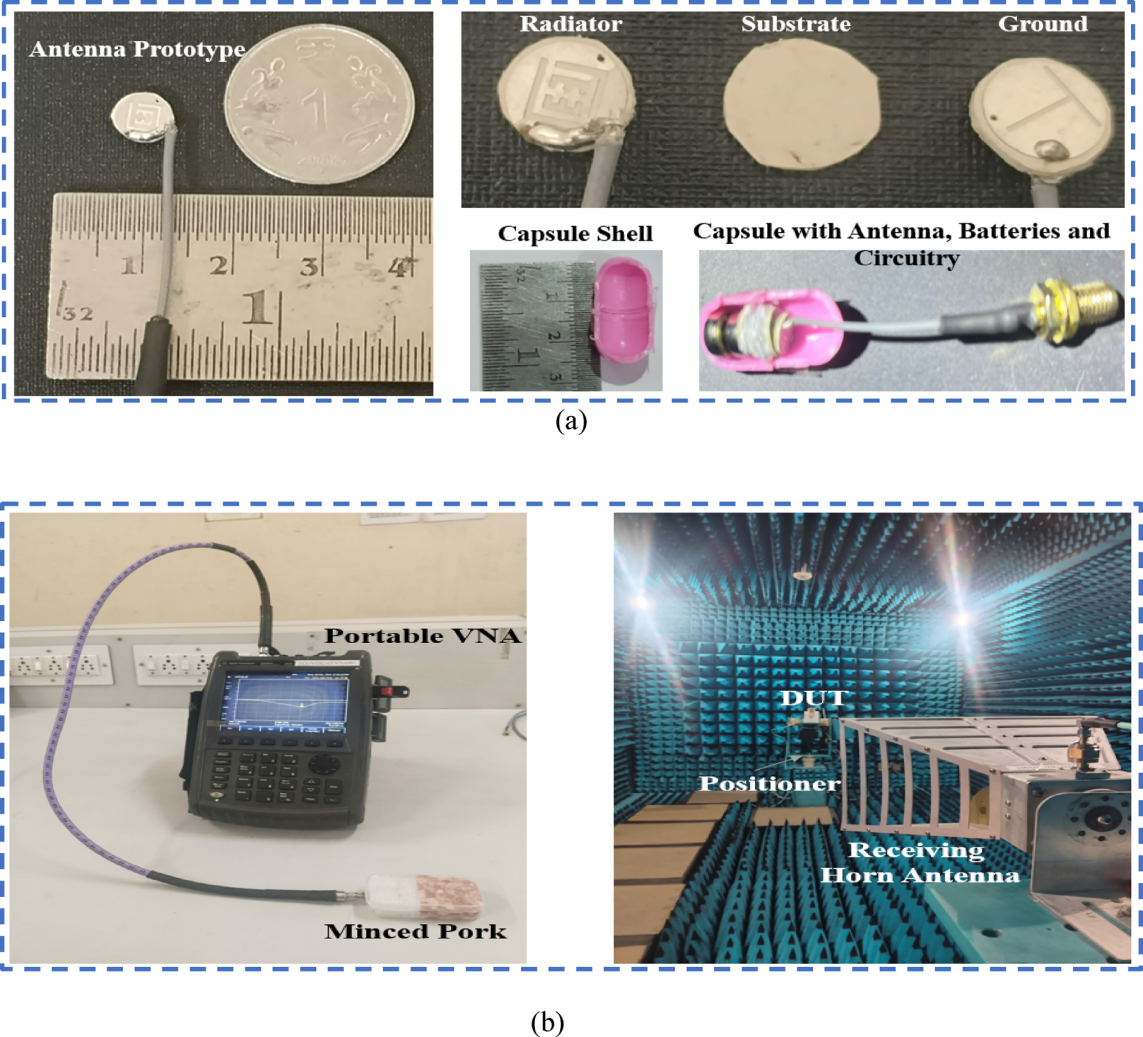
(**a**) Implantable antenna prototype with a capsule (**b**) Measurement setup using the VNA and anechoic chamber.

The antenna’s simulated performance aligned with the design goals. Following this, the antenna was precisely manufactured and enclosed within a biocompatible capsule, as depicted in Fig. 7. The capsule also integrates dummy components such as sensors, batteries, and a PCB. For measurement purposes, a coaxial cable passage hole is precisely designed to match the diameter of the cable conductor. To replicate a realistic test environment, the antenna reflection coefficient ($\:{S}_{11}$) and radiation performance were experimentally validated in a tissue-equivalent environment using minced pork, which simulates biological dielectric properties. We obtained minced pork (pig stomach) from a local commercial vendor; no animals were slaughtered specifically for this research. $\:{S}_{11}$ measurements were conducted via a vector network analyzer (Agilent N5247A).

**Fig. 8:**
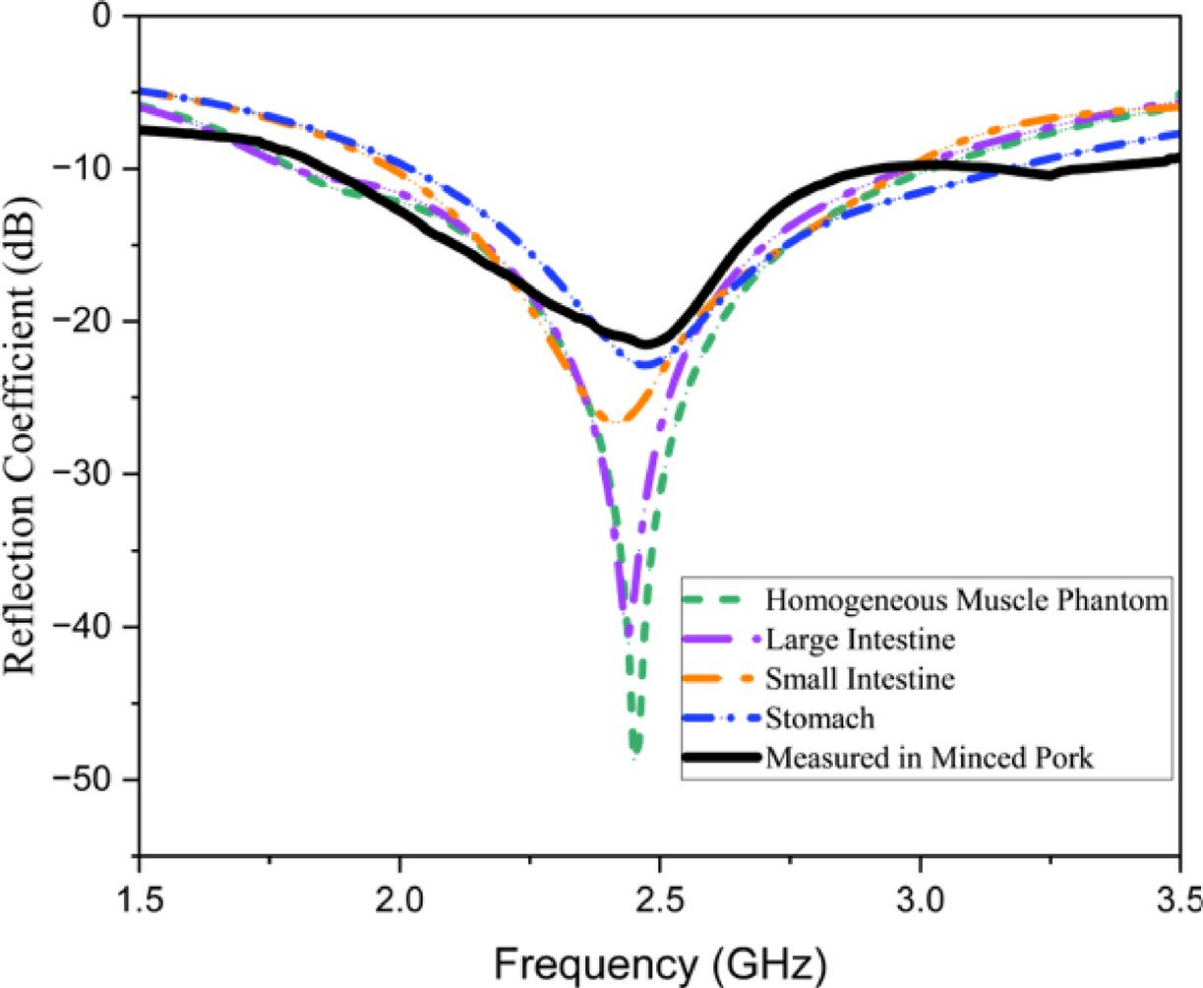
Reflection coefficients for various implant scenarios.

Figure 8 illustrates the proposed implantable antenna’s performance across multiple scenarios, demonstrating its superior effectiveness in each case. The 10 dB impedance bandwidths were 1220 MHz (1800–3020 MHz) for the homogeneous muscle phantom, 1170 MHz (1800–2970 MHz) for the large intestine phantom, 980 MHz (1900–2970 MHz) for the small intestine phantom, 1140 MHz (2030–3170 MHz) for the stomach phantom and 1050 MHz (1860–2910 MHz) for the minced pork. Figure 8 presents a comparison between the measured and simulated $\:{S}_{11}$ values for various scenarios. The experimental results verify the antenna’s effective operation in the 2.45 GHz ISM band. Minor differences between measured and simulated data are likely due to variability in the dielectric properties of the heterogeneous tissues used during testing. Additionally, fabrication tolerances, including gaps between the substrate and the superstrate, may contribute to antenna performance variations.

**Fig. 9:**
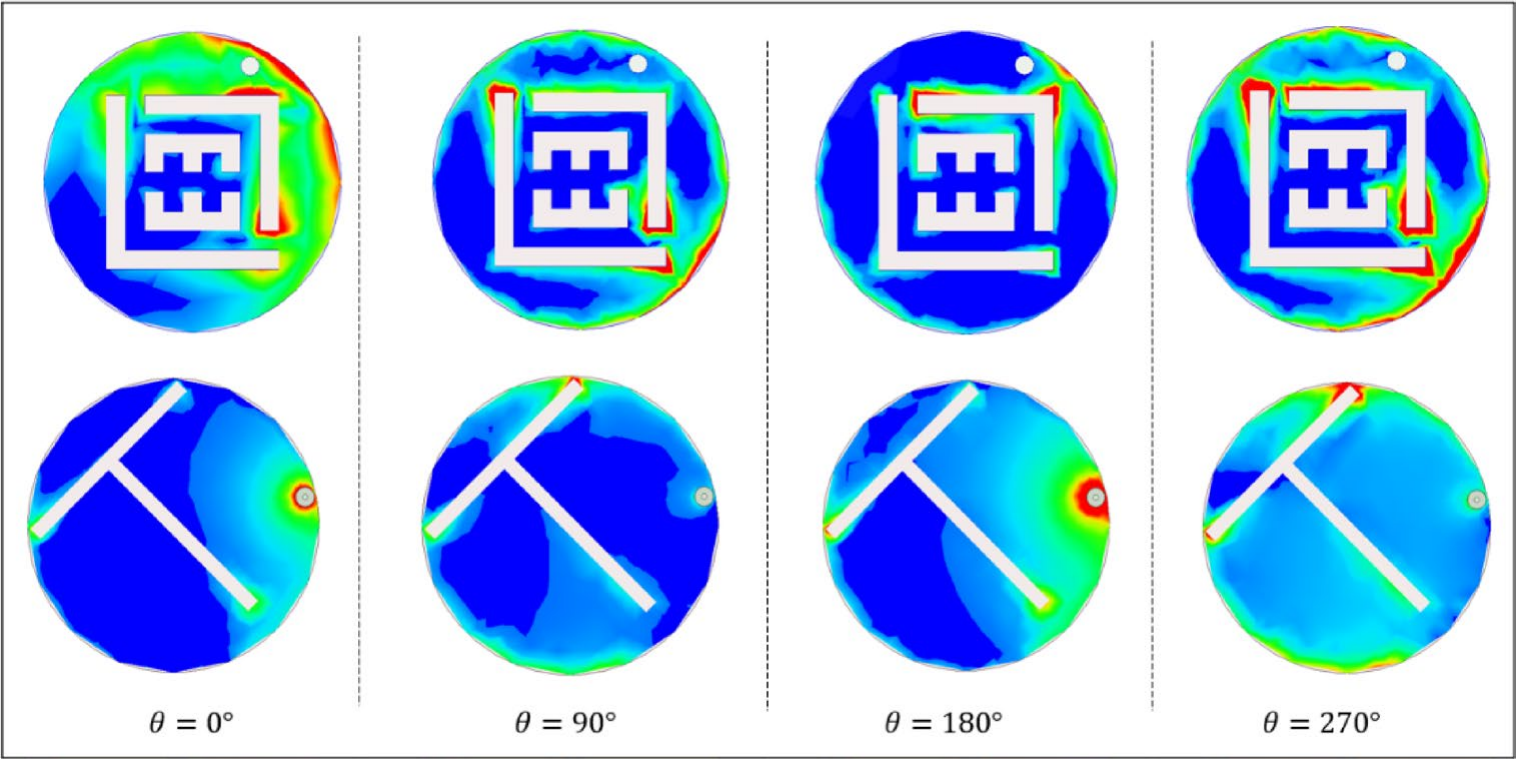
Current distributions of the proposed implantable antenna at various angles.

Figure 9 illustrates the current delivery on the antenna at four phase angles. At 0° and 180°, the current concentrates around the feed point with opposite polarity, signifying a phase inversion while maintaining the same electromagnetic behavior. At 90°, the current travels from the feed toward the groundplane shorting pin, highlighting enhanced coupling between the radiator and ground. This interaction effectively lengthens the current path, and contributes to the antenna’s resonance characteristics and overall impedance matching. Similarly, at 270°, the current distribution mirrors the behavior observed at 90°, but with an inverted polarity. This reversal is crucial in maintaining the antenna’s functionality, ensuring stable operation within the designated frequency ranges. The observed current patterns highlight the role of the shorting pin and radiator in shaping the antenna’s electrical performance, particularly in achieving efficient radiation and maintaining compactness.

**Fig. 10:**
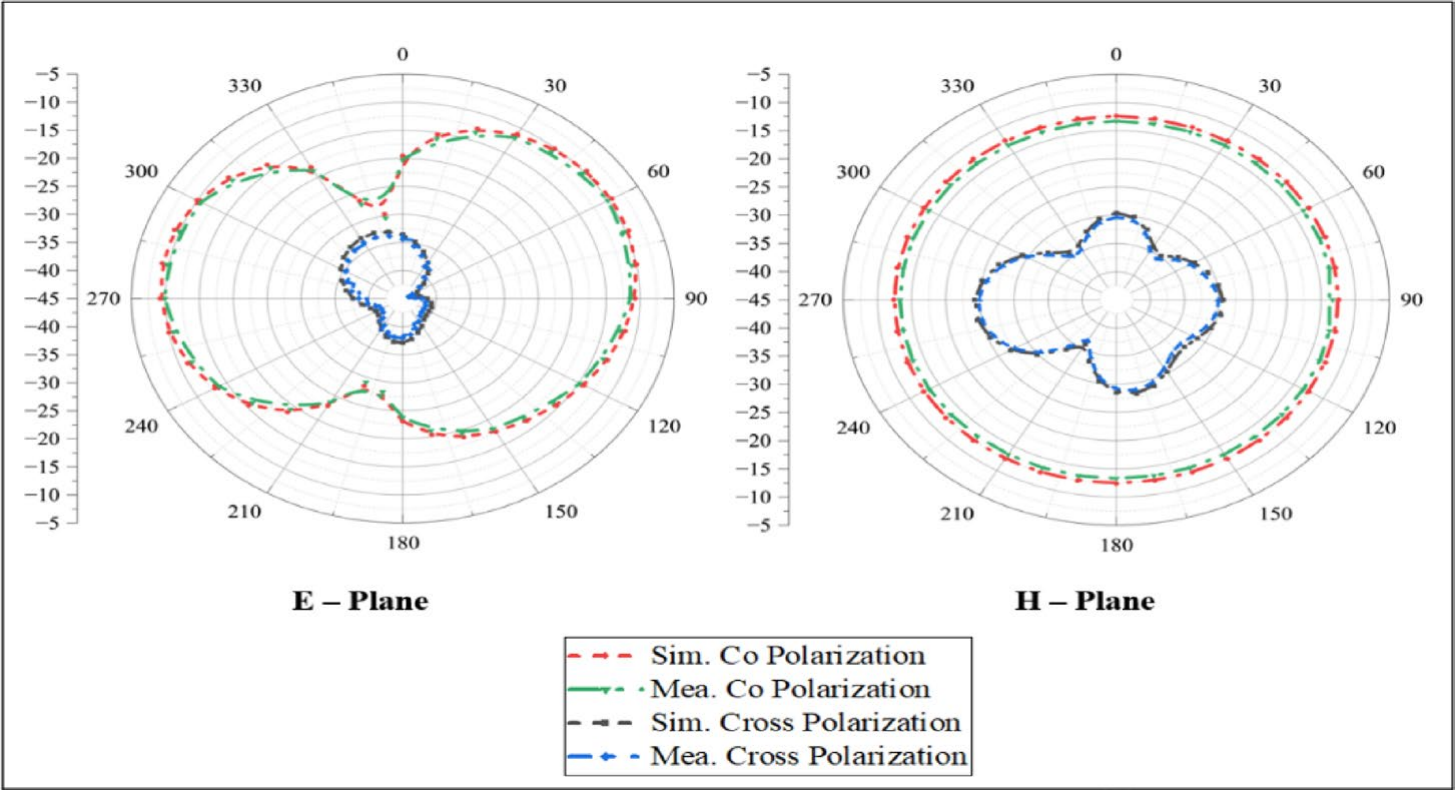
Co and cross polarizations of the proposed implantable antenna at 2.45 GHz.

Figure 10 compares the simulated and measured radiation patterns of the proposed antenna at 2450 MHz. The measurements were carefully conducted in an anechoic chamber to minimize external interference and ensure precise data collection. In the experimental setup, the antenna was positioned at the center of a container filled with minced pork, serving as a realistic tissue substitute. This arrangement not only facilitates the handling process but also replicates the conditions the antenna encounters within the human tissue. The test implantable antenna and receiving horn antenna were positioned 5 m apart to maintain the far-field measurement conditions. During the tests, one antenna was terminated with a 50 Ω load to preserve impedance matching, whereas horn antenna was associated with spectrum analyzer to record the radiation characteristics. To map the radiation pattern thoroughly, the test antenna was rotated in 5° increments while the horn antenna remained stationary. This systematic rotation revealed that the antenna exhibited an omnidirectional pattern at 2.45 GHz, a critical feature for ensuring consistent and reliable communication regardless of the capsule’s direction within the gastrointestinal tract. In simulations performed using a realistic human model with an antenna positioned inside the stomach, the peak realized gain was − 22.4 dBi at 2.45 GHz. These simulation results closely correspond to the experimental measurements, which yield peak gain values of approximately − 20.8 dBi at 2.45 GHz.

**Fig. 11:**
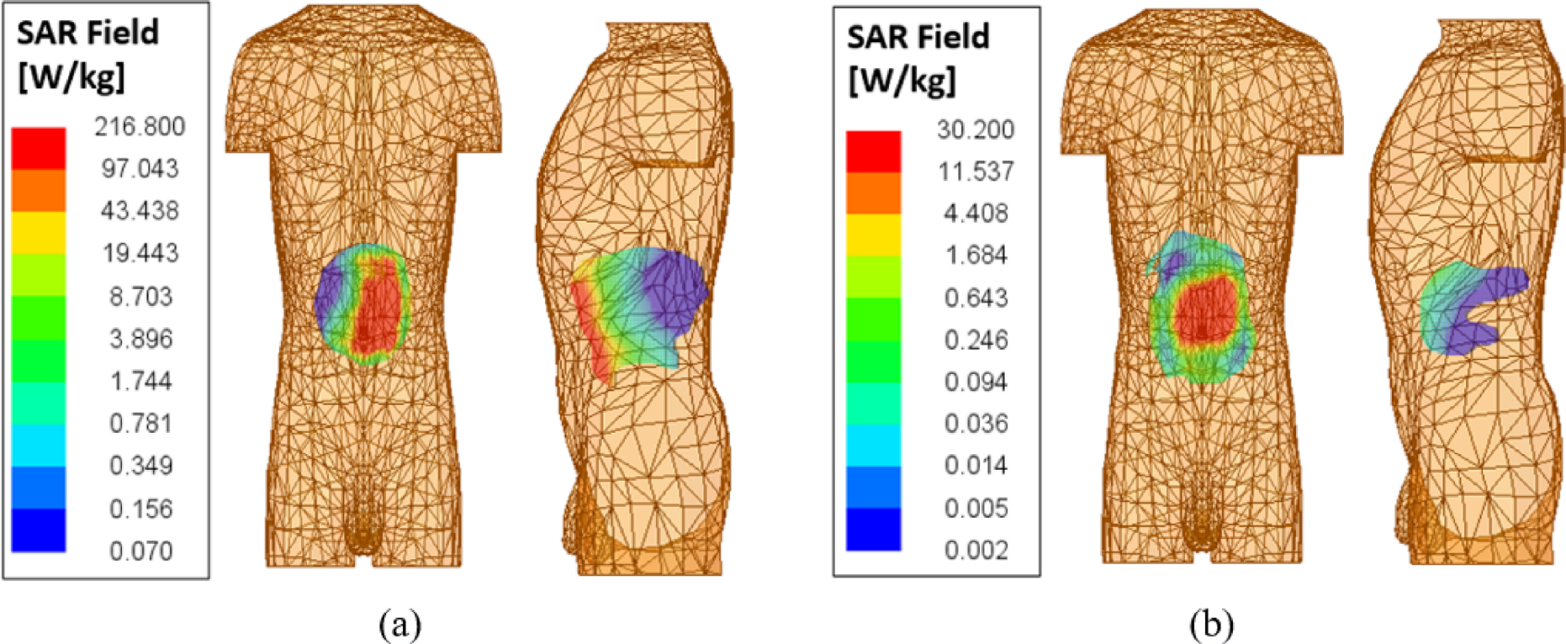
SAR distribution of the proposed implantable antenna at 2.45 GHz (**a**) 1 – g (**b**) 10 – g.

Assessing the Specific Absorption Rate (SAR) is crucial to confirm patient safety in the use of in-body wireless capsule endoscopy (WCE). Based on the updated IEEE C95.1-2019 guidelines, the SAR limit for a 10-g average is set at 2 W/kg^31^. For evaluation purposes, a human torso phantom containing an implanted capsule (depicted in Fig. 2) is utilized, with all simulations conducted using an input power of 1 W at the antenna port. Table 3 presents detailed SAR peak values at the designated frequencies, covering both 1-g and 10-g averages across various phantom models. The simulation findings show that the maximum 1-g and 10-g average SAR values at 2.45 GHz are 216.8 W/kg and 30.2 W/kg, respectively, it is substantially exceeding the IEEE safety threshold, as illustrated in Fig. 11. The maximum permissible input power for implantable antennas is 25 µW, which is suggestively less than the input power used in this design. The goal of the simulation is to verify that the SAR values of the implanted antenna comply with regulatory standards. For human exposure safety, the allowable power is 7.38 mW for 1 gram of tissue and 66.22 mW for 10 g of tissue. Based on the analysis, the SAR values align with the limits established by IEEE standards. On the basis of findings, the maximum allowable input power levels were calculated and their values are presented in Table 3. Notably, these values are considerably lower than the typical power levels used for implants as specified by the ITU-R SM.2153-8 standard^32^. Overall, the results confirmed that the proposed antenna design is appropriate and safe for operation within the human body.

**Table 3:** Average SAR and determined power for various implant scenarios.

Frequency (MHz)	Phantom model	SAR (W/kg)		Max. allowed power (mW)	
		C95.1–1999	C95.1–2005	C95.1–1999	C95.1–2005
2450	Muscle	284.7	61.8	5.62	32.36
	Large intestine	202.3	44.8	7.90	44.64
	Small intestine	208.4	49.6	7.67	40.32
	Stomach	214.5	50.8	7.46	39.37
	Human torso	216.8	30.2	7.38	66.22

Table 4 clearly shows that the proposed antenna design offers several advantages over current implantable antenna models. The implantable antennas listed in the table are larger in size; however, they exhibit significantly lower impedance bandwidths and gains than those of the proposed design. In contrast, the proposed structure features an upfront design, enhanced gain, a broader bandwidth, and improved radiation performance. The proposed design is also compatible with integrated circuits, thereby reducing fabrication complications.

**Table 4:** Comparison of the proposed work with their recent studies.

Ref/ Year	Dimensions (mm)	Frequency (MHz)	Gain (dBi)	Bandwidth (MHz)	SAR(W/kg)		Dielectric material	Implant depth (mm)
					1 – g	10 – g		
^23^/ 2020	$\:7\:\times\:7\:\times\:1.6$	915	-35.5	300	-	-	Silicon	-
^24^/ 2021	$\:6.5\times\:6.5\times\:0.05$	915, 2450	-28.2, -24.5	123.5, 154.4	420.3, 233.2	45, 27.7	ULTRALAM 3850HT	50
^25^/ 2024	$\:\pi\:\:\times\:\:{5}^{2}\:\times\:1.28$	2450	-31.9	150, 160	137.9	67.2	Rogers 3010	90
^26^/ 2019	$\:\pi\:\:\times\:\:{5}^{2}\:\times\:0.635$	2450	-26.4	520	712.1	-	Rogers 3010	4
^27^/ 2024	$\:\pi\:\:\times\:\:{4.9}^{2}\:\times\:0.635$	2450	-24.6	150	331	45.58	Rogers RO 6010	-
^28^/ 2024	$\:\pi\:\:\times\:\:{4}^{2}\:\times\:0.889$	2450	-26.7	120	168	-	Rogers RO 6010	30
^29^/ 2022	$\:2.6\times\:3\times\:0.381$	2450	-9.7	148	596.33	-	Rogers RO 3010	25
^30^/ 2024	$\:\pi\:\times\:{3}^{2}\times\:0.254$	2450	-24.5	400	274.6	29.7	Rogers RT/Duroid 5880	50
This work	$\pi \times 3^{2} \times 0.254$	2450	-20.8	1050	216.8	30.2	Rogers RO 3010	75

### Link budget analysis

The primary purpose of the antenna is to communicate data captured by the implant to a data acquisition device. The communication performance of the antenna must be assessed before it can be integrated into a practical implant. This is achieved through a link budget (LM) analysis that considers factors such as free space losses, path loss exponent, antenna mismatches, cable loss, and shadowing effects^33^. Reliable communication necessitates that the LM remains above 0 dB^34^; hence, a 20 dB LM is targeted in this study to ensure uninterrupted data transfer. A theoretical evaluation of the antenna’s communication capabilities was carried out using the Friis equations, as described in^35^. In this analysis, the proposed antenna functioned as the transmitting element, while a horn antenna operated as the receiving element. Data was collected by varying the distance between the transmission and reception antennas, with the implantable antenna functioning at a transmitted input power of − 16 dBm (25 µW).

The link margin is calculated as follows,

1 $$\:Link\:margin\:\left(dB\right)=Available\:Power\:\left({R}_{a}\right)-Required\:Power\:\left({R}_{r}\right)$$

The required antenna power ($\:{R}_{r}$) is calculated as follows:

2 $$\:{R}_{r}=\:\raisebox{1ex}{${E}_{b}$}\!\left/\:\!\raisebox{-1ex}{${N}_{0}$}\right.+10{\text{log}}_{10}\left({B}_{r}\right)-{G}_{c}+{G}_{d}$$

Additionally, the antenna’s available power ($\:{R}_{a}$) is computed via the following expression:

3 $$\:{R}_{a}=\:{P}_{t}+{G}_{t}+{G}_{r}-{L}_{f}-{N}_{0}$$

**Fig. 12:**
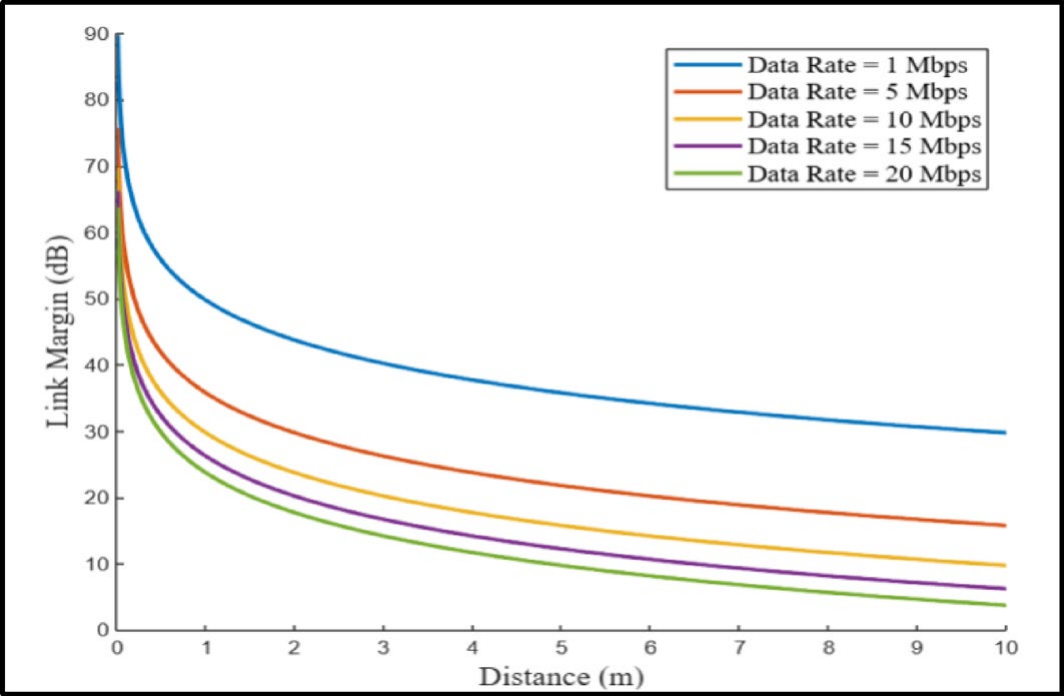
Link margin examination of proposed implantable antenna relative to the bit rate and transmission range.

In this framework, $$\:{R}_{r}$$ denotes the minimum power (in dB), with T representing the temperature (293 K), $$\:\raisebox{1ex}{${E}_{b}$}\!\left/\:\!\raisebox{-1ex}{${N}_{0}$}\right.$$ indicating the PSK modulation threshold at 9.6 dB, K denoting Boltzmann’s constant (1.38 × 10⁻²³), and B_r_ denoting the bit rate. Table 5 provides a summary of all the essential parameters used in the link budget analysis. In this study, five different bit rates: 1, 5, 10, 15, and 20 Mb/s were considered for the calculations at an operating frequency of 2.45 GHz. Figure 12 shows the transmission ranges corresponding to these data rates. The results indicate that the antenna reliably transmits data over distances exceeding 10 m with a 15 dB link margin at both operating frequencies. Moreover, at the ISMband frequency, it can sustain 20 Mb/s data rates beyond 10 m with the same margin. These findings highlight the antenna’s efficiency for biotelemetry applications.

**Table 5:** Constraints of link budget calculation.

Parameter	Value
Operating frequency $\:{f}_{0}$ (GHz)	2.45
Transmitter gain $\:{G}_{t}$(dBi)	-20.8
Transmitter power $\:{P}_{t}$ (dBm)	-16
Receiver antenna gain $\:{G}_{r}$(dBi)	2.15
Distance (m)	1–10
Bit rate $\:\left({B}_{r}\right)$	1 Mbps, 5 Mbps, 10 Mbps, 15 Mbps, and 20 Mbps
Free space path loss	Distance dependent
Available power	Distance dependent
Coding gain $\:{G}_{c}$ (dB)	0
Fixing deterioration $\:{G}_{d}$ (dB)	2.5

## Conclusion

This article presents the development of a compact and wideband implantable antenna system specifically designed for wireless capsule endoscopy (WCE). The design incorporates several innovative features such as a shorting pin, slots in the radiator, and a defective ground structure, to achieve both miniaturization and wideband functionality. With an overall volume of 7.18 mm³, the antenna functions within the ISM 2.4 GHz band, as confirmed by the simulation and experimental results using both a human body phantom and minced pork. Furthermore, a capsule device measuring 5.5 mm in radius and 26 mm in length was developed to evaluate the antenna’s performance in an environment that closely mimics real conditions. Despite being implanted at a considerable depth, the antenna achieved − 20.8 dBi gain at 2.45 GHz. Safety evaluation according to IEEE SAR standards shows a 10 g SAR of 30.2 W/kg at this frequency. Overall, this antenna design shows great promise as a viable option for implantable medical devices used in capsule endoscopy.

## Data Availability

All data generated or analysed during this study are included in this article.
